# Against Substitution

**Published:** 1901-06

**Authors:** 


### AGAINST SUBSTITUTION.

The Tennessee Legislature has passed the following act and the Governor has signed it; it is a law. The State of Texas should follow the example and at the next session pass a similar law.

An Act to prevent the substitution of any drug in filling physicians' prescriptions by druggists in the State.

SECTION 1. Be it enacted by the General Assembly of the State of Tennessee, That it shall be unlawful for any corporation, firm or person, or any combination or association of corporations, firms or persons engaged in the business of buying, compounding and selling drugs and medicines to substitute any drug or medicine in lieu or instead of that given to the patient by the physician on the face of his prescription.

SEC. 2. Be it further enacted, That it shall be unlawful for any agent or employe of such person, firm or corporation or association or combination of persons, firm or corporations engaged in the business of buying and selling drugs in this State to substitute any medicine for the specific medicine mentioned in the physician's prescription.

SEC. 3. Be it further enacted, That any person, firm or corporation violating the provisions of this act, or aiding or abetting the violations of the same shall be guilty of a misdemeanor and upon conviction shall be fined not less than $25 nor more than $100 for each and every offense.

SEC. 4. Be it further enacted, That this act take effect from and after its passage, the public welfare requiring it.

